# Acquired Deforming Hypertonia in Afro-Caribbeans: A Cross-Sectional Analysis in Long-Term Care Units

**DOI:** 10.3390/jcm14041192

**Published:** 2025-02-12

**Authors:** Nicolas Kerjean, Rishika Banydeen, Bertrand Glize, Michel Bonnet, Patrick Rene-Corail, Maturín Tabue Teguo, Moustapha Dramé, Patrick Dehail, Jose-Luis Barnay

**Affiliations:** 1 Centre Hospitalier Universitaire de Martinique, 97200 Fort-De-France, France; nicolas.kerjean@chu-martinique.fr (N.K.); rishika.banydeen@chu-martinique.fr (R.B.); michel.bonnet@chu-martinique.fr (M.B.); patrick.rene-corail@chu-martinique.fr (P.R.-C.); tabue.maturin@gmail.com (M.T.T.); moustapha.drame@chu-martinique.fr (M.D.); 2 Service de Médecine Physique et Réadaptation, Pôle de Neurosciences Cliniques, CHU de Bordeaux, 33000 Bordeaux, France; bertrand.glize@chu-bordeaux.fr (B.G.); patrick.dehail@chu-bordeaux.fr (P.D.)

**Keywords:** acquired deforming hypertonia, contracture, prevalence, Caribbean, long-term care units, insular environment

## Abstract

**Background:** Osteoarticular deformities or contractures in institutionalized elderly individuals, described as acquired deforming hypertonia (ADH), have a multifactorial origin. The reported prevalence of ADH in French Caucasian patients in long-term care units (LTCUs) is 25.6%. To date, ADH in the Caribbean population has never been studied. We aimed to assess the prevalence and characteristics of ADH in such a population. **Materials and Methods:** This was a cross-sectional observational study of a French Caribbean population in Martinique in which patients aged 75 years or older were institutionalized in LTCUs during the study period. Data extraction from the medical files of eligible LTCU patients was conducted to assess the prevalence, clinical characteristics, and impact of ADH on patients’ daily care. The assessments were performed collaboratively between the patients’ geriatric team and a PM&R physician. **Results:** In total, 81 patients were included, with an ADH prevalence of 77.8%. Reported ADH was bilateral (86%) or multiple (66% of patients had ≥5 ADH) and was responsible for major alterations in terms of hygiene, dressing, pain, and skin damage. ADH patients had a high level of dependence (GMP = 924), and this level of dependence was significantly associated with the presence of at least one ADH (*p* < 0.001) regardless of prior disease. **Conclusions:** The incidence of ADH in our Caribbean population seems twice as high as that in Caucasian patients, underlining the necessity for this nosological framework to be better recognized, particularly in an insular context. Local campaigns for the prevention and recognition of ADH must be considered, and targeted multidisciplinary protocols need to be established for adapted care in all institutions receiving elderly people.

## 1. Introduction

An aging world population is a reality that must be considered in both hemispheres [1,2,3,4]. With advancing age, dependency often occurs [4,5,6,7,8]. In France in 2015, 10% of the population over the age of 75 lived in institutions, 80% of whom were in residential or care facilities for dependent older people, mainly due to a loss of autonomy linked to the increase in neurodegenerative pathologies and familial isolation [4,5,6,7,8]. While Martinique has a similar health system as mainland France, notable differences exist: less medical infrastructure and equipment, a lower density of medical resources, and greater social deprivation.

The specific characteristics of the French population living in the Caribbean tend to increase the prevalence and impact of pathologies. Those with no standardized care recommendations are more sensitive to environmental factors and weaknesses in the healthcare system, particularly vulnerable and dependent individuals. By 2030, Martinique is projected to be the oldest region in France, with 40% of its population aged 60 years and older [2].

In institutionalized elderly individuals, osteoarticular deformities or contractures are frequent phenomena of multifactorial origin. In 2014, the French team of Dehail et al. proposed a new nomenclature, regrouping the latter under the term “acquired deforming hypertonia” (ADH), in consultation with a committee of experts consisting of geriatricians, physical medicine and rehabilitation (PM&R) physicians, and orthopedic surgeons [9]. ADH is defined as “any joint deformity with reduced amplitude and increased resistance to passive mobilization, regardless of the cause, and resulting in functional discomfort, or any other limitation in daily life activities” [9,10,11,12,13,14,15,16,17,18,19].

ADH management in elderly institutionalized individuals varies according to patient profile (bedridden, ambulatory, or partly autonomous) and ranges from light posture and installation to toxin injection, motor or nerve selective blockade, or tailored frailty-adapted surgery (needle tenotomy, casting, or light orthopedic surgery). The global strategy of organized medical care is to promote passive movement, occupational strategy, and physical activity to constantly induce movement and avoid or prevent ADH installation [10,20,21,22,23,24,25].

The prevalence of ADH in institutionalized patients varies between 20% and 75%. In mainland France, which is majority Caucasian, the prevalence is estimated to be 22% (25.6% in long-term care units (LTCU) and 20.2% in other residential facilities), with a significant impact of ADH on the quality of life and care load of patients [10,26]. However, to date, there is a dearth of data on the prevalence and characteristics of ADH in non-Caucasian patients, such as Caribbean patients. In the present study, we aimed to assess the prevalence of ADH, clinical characteristics, and impact on daily patient care in a predominantly Caribbean insular population aged 75 years or older and institutionalized in long-term care units (LTCUs).

## 2. Materials and Methods

### 2.1. Study Context, Design and Population

The present exploratory observational study involved the three LTCUs of the French Caribbean territory of Martinique; the LTCUs are affiliated with the sole University Hospital of the island. For study purposes, from 6 August to 29 September 2020, a review of the medical records of all institutionalized patients aged 75 years or older was conducted to identify existing deformations that corresponded to the ADH definition. Patient exclusion criteria were the existence of an episode of acute alteration of health status, uncontrolled chronic pain with an identified origin different from that of ADH, and the existence of an evolving neoplastic process in the palliative stage. To ensure the most reliable possible collection of cases of ADH, the healthcare teams were sensitized by the rehabilitation physician. Additionally, as in previous prevalence studies, patients with active pathologies or joint diseases were excluded to avoid overestimating the prevalence of deformities.

This study was approved by the institutional review board of the University Hospitals of Martinique (IRB reference number: 2020/082, on November 2020). All patients were managed in accordance with the amended Declaration of Helsinki. Informed consent from the patients or patients’ legal guardians/next of kin was obtained prior to study inclusion in accordance with current legislation and institutional requirements.

### 2.2. Study Data

Demographics and clinical data were collected. The level of dependence of patients was evaluated by their referring geriatrician at each participating LTCU according to their GIR (groupe iso-ressource: Scale of level of loss of autonomy of an elderly person, rated from 6 (fully autonomous) to 1 (bed-ridden)) and severity (measured by the AGGIR grid (autonomie gérontologique groupes isos ressource, GIR measurement chart)). The average dependency level of each LTCU was evaluated by the weighted average GIR (GMP), which corresponds to the average dependency level of their residents [27].

Clinical diagnosis of ADH was made according to the definition of Dehail et al. [9] For each study participant, ADH diagnosis was conducted by a PM&R physician. The clinical features of ADH patients were described according to their topography, number of patients per individual, or type. The topographical descriptions of the upper limb were shoulder adductum, elbow flessum, wrist flessum, and/or finger claw. For the lower limb, the following locations were described: adductum and/or flessum of the hip, flessum of the knee, equinus and/or varus of the ankle, and claw of the toes. Disorders of spinal statics in the frontal and/or sagittal plane of the cervical spine, attributable to ADH, were also identified, such as cervical deformation.

The impact of ADH on the daily care of patients was assessed collaboratively with the nursing teams of each LTCU using a standardized and validated ad hoc evaluation grid [9]. For the upper limbs, the following data were collected: difficulty grasping directly attributable to ADH; difficulty in hygiene, nursing, and dressing; the presence of skin complications (mycosis, pressure sores); and spontaneous or provoked pain during passive mobilization according to the Bourreau behavioral scale [9]. The presence of difficulties walking and/or transferring directly attributable to ADH was recorded, as were difficulties in hygiene and nursing care, dressing and shoeing, problems settling in a chair, skin complications (mycosis, pressure wounds), and spontaneous or provoked pain during passive mobilization. For axial ADH patients, the collected information concerned discomfort during hygiene/nursing, bed positioning, standing and/or transferring, walking, spontaneous or provoked pain during passive mobilization, and skin complications (mycosis, pressure wounds).

### 2.3. Statistical Analysis

For all descriptive and inferential analyses, the assumption of a normal data distribution was analyzed. The means and standard deviations are reported for normally distributed variables, and the medians and interquartile ranges (IQRs) are reported for nonnormally distributed variables. Categorical variables are presented as absolute values and percentages. The following tests were used for group comparisons, when appropriate: Student’s *t* test, Wilcoxon–Mann-Whitney test, chi-square test, and Fisher’s exact test. The prevalence of ADH, expressed per 100 persons with a 95% confidence interval (CI), was determined. Patient characteristics were also described, and ADH presence according to patient level of dependence and medical history was analyzed. Moreover, the association between the number of ADH patients and the number of difficulties encountered by the caregivers during patients’ daily care was assessed via the use of a linear regression model. All the statistical analyses were performed using SPSS Statistics for Windows, version 27.0 (SPSS, Inc., Chicago, IL, USA), with *p* values < 0.05 considered to indicate statistical significance.

## 3. Results

**Figure 1:**
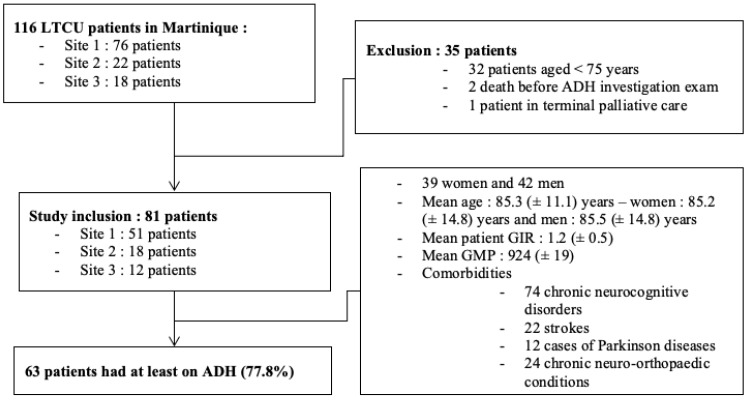
Study flow chart.

A total of 116 eligible patients were identified during the study period (Figure 1). Concerning the general data, 39 women and 42 men were examined. Participants ranged in age from 75 to 100 years, with an average age of 84.9 years (84.5 for men and 85.2 for women). In terms of previous history, 22 had had at least one stroke, 12 had Parkinson’s disease, 74 had chronic neurocognitive disorders, and 24 had other chronic neuro-orthopedic conditions.

Only 81 patients were ultimately included in the study, with 63 participants presenting with at least one ADH, accounting for a prevalence of 77.8% IC95% (68.7–86.8%).

The mean age of the study participants was 85.3 ± 11.1 years, and the female:male sex ratio of patients with at least one ADH was 1.1 (33 women and 30 men). Overall, regarding medical history, 22 patients had suffered at least 1 stroke, 12 had Parkinson’s disease, 74 had chronic neurocognitive disorders, 24 had other chronic neuro-orthopedic conditions, and 2 had a previous history of arbovirus infections (Dengue, Chikungunya, Sika). The average GIR of the study participants was 1.2 ± 0.5, while the average GMP, which was related to the number of participants according to their respective LTCU, was 924 ± 19.

ADH was bilateral in 83% of the patients (*n* = 52), and the topographical distribution of the patients is shown in Table 1. When ADH was present, several joints were affected simultaneously: 5% of the diagnosed patients had a single ADH, 66% had at least 5 ADH, and more than one-third presented more than 10 ADH at the time of study inclusion. Lower limb and upper limb involvement was observed in 89% and 81%, respectively, of the ADH patients. Elbow involvement was most frequently reported for the upper limbs (73%), while knee involvement was the most common for the lower limbs (68%). Axial ADH was associated with an average of 9.5 ± 3.3 other ADH genes in the same individual.

There was a significant association between the presence of at least one ADH and the dependency score assessed by the GIR grid, with 85% of the GIR patients having an ADH, 55% having a GIR 2, and none having a GIR 3 (*p* < 0.001). Similarly, there was a significant association between the average dependency level of individuals in each LTCU (estimated by the GMP) and the presence of at least one ADH in their institutionalized patients (*p* = 0.007). However, there were no significant associations between the presence of ADH and dementia (*p* = 0.67, IC95% (0.5669–0.7730)), stroke (*p* = 0.08, IC95% (0.205–0.1394)), Parkinson’s disease (*p* = 0.21 IC95% (0.1207–0.2992)), or past arbovirus infection (*p* = 0.44 IC95% (0.5549–0.8087)). The difficulties encountered by LTCU caregivers for patients with ADH are described in Table 2.

Hygiene and/or nursing care, as well as dressing, were the main difficulties encountered for upper and lower limb disorders. Lower limb impairment was frequently associated with discomfort when standing up and sitting in a chair. Skin damage was more frequent in the case of damage to the upper limbs. Axial damage had a significant impact on hygiene and/or nursing care, mainly in relation to bed or chair positioning. However, this procedure appeared to be less painful than peripheral damage, with only one out of two axial ADH patients being painful. Care-induced pain was important for all types of ADH. The more distal the affected joint was, the greater the observed risk of skin complications. Maceration-type injuries were more frequently found on the wrists/hands (85%), while pressure wounds were mainly observed on the ankles (52%) and feet (54%) (Table 2).

**Figure 2:**
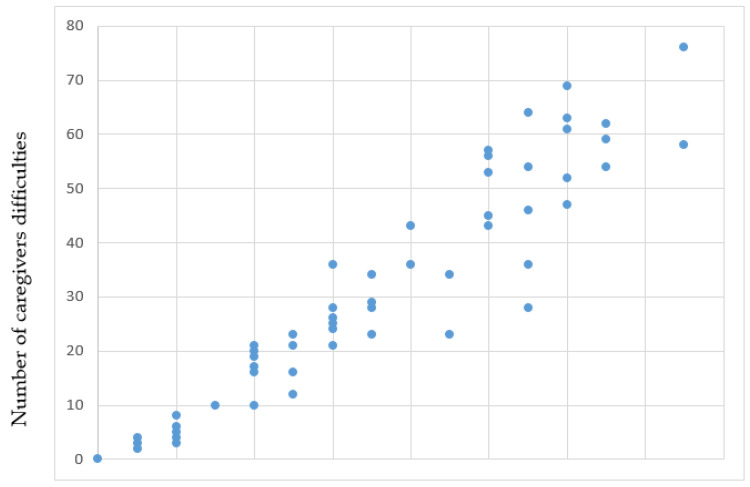
Association between acquired deformation hypertonia (ADH) presence and caregiver-reported difficulties (linear regression).

Furthermore, the number of caregiver difficulties was significantly associated with the number of ADH disorders reported by a patient (R^2^ = 0.883, *p* value < 0.001) (Figure 2).

## 4. Discussion

To the best of our knowledge, our study is the first to be conducted in a predominantly Caribbean population to provide a prevalence estimate of osteoarticular deformities, called ADH, in the Caribbean region. We report a high prevalence of ADH (77.8%), IC95% (68.7–86.8%), for a rather homogeneous population in rehabilitation care with a higher level of dependence.

Previous reports have described an ADH prevalence ranging from 24% to 88% in other populations [9,11]. As such, the observed prevalence of 77.8% in our Caribbean study population is among the highest rates described thus far. In comparison to pioneering work [9], our patient inclusion modalities differ, as ADH diagnosis was carried out by a PM&R physician in direct collaboration with on-site physicians at each of the three participating LTCUs [9]. This might have contributed to optimized ADH diagnosis due to greater expertise in identifying neuro-orthopedic deformities and resulting in a greater prevalence of ADH.

Moreover, the level of patient dependence in our study was high, with an average GMP of 924 years compared with 854 years in mainland France [9], with a similar approximate mean age of 85 years. We also observed a significant statistical relationship between the level of dependence of the participating institutions and the risk of ADH onset: the greater the level of dependence, the greater the risk of developing ADH (R^2^ = 0.998, *p* = 0.007). The late use of specialized institutions for patients with more comorbidities in the territory is consistent with this observation of a higher ADH incidence, in comparison to the 25.6% prevalence found by Dehail et al. [9]. The specific characteristics of this population could partly explain this over-prevalence. Medical data show a lower rate of hospitalization, a higher prevalence of chronic diseases and their complications, and an earlier onset of dementia. These are medical factors that increase the risk of developing ADH. Other socio-economic factors also impact on the risk of developing this phenomenon: a higher poverty rate than in mainland France, a weaker medical demography, and greater difficulties in accessing care [1]. It therefore seems difficult to implement a prevention and early management approach to this phenomenon, which means that institutionalized patients run the risk of increasing the prevalence of ADH.

ADH management suffers from a lack of knowledge by medical and paramedical teams, who also manifest a certain fatalism toward the condition: 3 out of 4 practitioners considered ADH irreversible, with frequent therapeutic abstention [9]. This was also observed among caregivers during the present research, with the latter being totally unaware of ADH and its possible multidisciplinary management. We observe, in the literature, a lack of consensus on the use of the ADH term for identifying contracture and joint deformations in the dependent elderly population [26,28,29,30,31,32].

Hence, a minimal information campaign for caregivers in geriatric institutions is likely needed [9,10]. Many caregivers still use the term “contracture” in elderly populations and seemingly ignore the comprehensive definition, with the resulting consequences being less recourse to a multidisciplinary therapeutic arsenal with a tailored collaborative approach by PM&R physicians, geriatrists, and trained orthopedic surgeons with regard to the patient’s level of frailty.

We further observed a scarcity of paramedical staff in the three participating LTCUs, which is in line with the general situation in all public health care institutions on the island. In the rehabilitation field, we report one physiotherapist for more than one hundred patients and a quasisystematic absence of occupational therapists—far too few to allow for adequate prevention and therapeutic management in LTCUs [27]. In this context of poor density of specialized personnel and care rationalization, interactions between patients and caregivers limit themselves to the conduct of routine activities, which is counterproductive to the development of patient autonomy [21,22]. It has been proven that the absence of stimulation does not allow the maintenance of functional skills and accelerates the loss of autonomy in fragile and dependent patients [28,29,30,33,34].

A notable strength of the present study is the exhaustive recruitment of all patients aged 75 years and older who were institutionalized in the three LTCUs of the island. However, the observational nature of the present study did not allow for the use of an ADH chronology. The observed high ADH prevalence in our Caribbean population must also be considered in light of the present study’s methodology (diagnosis by a PM&R physician) and the context of insularity. However, there are a number of limitations to this study. First, the small number of participants means that the results cannot be generalized, and the statistical results lack power.

Methodological changes in the way ADHs are counted in the study centers reduce the possibility of comparing populations, which in turn reduces the possibility of comparing results to objectify the population differences suggested in this manuscript.

Furthermore, even if the non-exclusion criteria were defined in the same way as in previous studies in this area, the small size of this study means that there is a risk of underestimating prevalence. It is also important to note that the USLD population, in the medico-socio-economic and cultural context described above, may also have induced a change in prevalence due to the risk of concentrating the most vulnerable patients

The geographical arrangement induced by the latter negatively impacts the healthcare system with regard to less equipment, a lower density of medical and paramedical staff resources, and greater social deprivation. When combined, these different elements might induce a greater handicap [10,30].

Global healthcare based on the one health concept can positively impact health strategies and provide more effective results by facilitating the implementation of cultural and societal specificities among nursing teams to prevent or reduce the impact of ADH through the use of personalized occupational strategies. Currently, the notion of “practicing care” in an institution must be replaced by “taking care” of elderly people: “care” before “cure” [21,35]. The results presented in this article are difficult to generalize, as they concern a captive, small, and highly vulnerable population. However, we can build on these results to develop more effective prevention strategies to avoid these unfavorable developments in dependent elderly people. We would also benefit from studies on a less dependent population in retirement homes, or even on non-institutionalized subjects losing their autonomy, by carrying out longitudinal studies to gain a better understanding of this phenomenon and its consequences.

## 5. Conclusions

The high prevalence of acquired deforming hypertonia (ADH) in Caribbean patients aged 75 years and older in long-term care units of an insular territory raises the question of its recognition, management, and prevention, particularly in insular low-resource settings. Our study results advocate for the importance of developing local and global strategies enriched by nursing knowledge, occupational therapy, physical activity, and rehabilitation. To improve the ability of healthcare to provide care for patients with ADH, teams must focus on global care, including strategy prevention, early in-home care, and adaptation occupational strategies at all institutions for elderly people with autonomy loss. This study is only a first step in this direction and needs to be extended to other Caribbean territories, representing approximately 48 million inhabitants. Additionally, the importance of ADH or joint contracture impact on patients’ quality of life should be measured, as should the necessary recognition and consideration of ADH for tailored patient care and its resulting medico-economic impact.

## Figures and Tables

**Table 1:** Acquired deformation hypertonia (ADH) distribution in the upper and lower limbs in elderly patients aged 75 years or older institutionalized in long-term care units in a predominantly Caribbean environment (*n* = 63).

Topography	Unilateral (*n* = 11)	Bilateral (*n* = 52)	Total (*n* = 63)
Upper extremity involvement	14 (22)	37 (59)	51 (81)
Shoulder abduction defect	8 (13)	32 (51)	40 (63)
Elbow flessum	11 (17)	35 (56)	46 (73)
Flessum of the wrist and/or finger claw	5 (8)	27 (43)	32 (51)
Lower limb involvement (overall)	7 (11)	49 (78)	56 (89)
Hip abduction defect and/or flessum	1 (2)	34 (54)	35 (56)
Knee Flessum	5 (8)	38 (60)	43 (68)
Foot equinus and/or varus	4 (6)	32 (51)	38 (60)
Toe claw	2 (3)	11 (17)	13 (21)
Axial damage to the cervical spine	-	-	36 (57)

Values are presented as absolute numbers (%).

**Table 2:** Acquired deformation hypertonia (ADH) consequences for caregivers’ reported difficulties during patients’ daily care.

Type of Impact	Upper Limb			Lower Limb				Cervical Spine (*n* = 36)
	Shoulder Adductum (*n* = 40)	Hip Adductum or Flessum (*n* = 35)	Wrist or Finger Flessum (*n* = 32)	Hip Adductum/Flessum (*n* = 35)	Knee Flessum (*n* = 43)	Foot Equinus and/or Varus (*n* = 38)	Toe Claw (*n* = 13)	
Hygiene or nursing care	33 (83)	30 (87)	28 (88)	30 (87)	36 (84)	31 (82)	9 (69)	24 (67)
Dressing ^a^/Shoeing ^b^/Sitting installation ^c^	32 (80)	31 (89)	25 (63)	31 (89)	36 (84)	29 (76)	9 (69)	27 (75)
Prehension difficulty ^a^/verticalization or transfer ^b,c^	17 (43)	27 (77)	15 (47)	27 (77)	29 (68)	25 (66)	8 (62)	4 (11)
Armchair installation for lower limbs				26 (74)	29 (67)	26 (68)	9 (69)	
Cutaneous complication								
Mycosis or Maceration	31 (78)	26 (74)	27 (85)	20 (57)	25 (58)	24 (63)	10 (77)	8 (22)
Pressure Wound	5 (13)	20 (57)	6(19)	12 (34)	16 (37)	20 (52)	7 (54)	11 (31)
Pain								
Spontaneous	4(10)	12 (34)	3 (9)	5 (14)	8 (19)	11 (29)	4 (31)	1 (3)
Induced	34 (84)	5 (14)	28 (88)	29 (83)	36 (84)	33 (87)	11 (85)	17 (47)

Values are presented as absolute numbers (%), ^a^: upper limb, ^b^: lower limb, ^c^: cervical spine.

## Data Availability

The original contributions presented in the study are included in the article, further inquiries can be directed to the corresponding authors.
